# Advances in the Molecular Modification of Microbial ω-Transaminases for Asymmetric Synthesis of Bulky Chiral Amines

**DOI:** 10.3390/microorganisms13040820

**Published:** 2025-04-03

**Authors:** Xinxing Gao, Qingming He, Hailong Chen, Wangshui Cai, Long Xu, Xin Zhang, Nianqing Zhu, Shoushuai Feng

**Affiliations:** 1 Jiangsu Key Laboratory of Chiral Pharmaceuticals Biosynthesis, College of Pharmacy and Chemistry & Chemical Engineering, Taizhou University, Taizhou 225300, China; gaoxinxing@tzu.edu.cn (X.G.); 123heqingming@163.com (Q.H.); xi_zhilang@126.com (H.C.); mg1424001@smail.nju.edu.cn (W.C.); xu_lon@163.com (L.X.); zhangxin2016@tzu.edu.cn (X.Z.); zhunianqing@tzu.edu.cn (N.Z.); 2 The Key Laboratory of Industrial Biotechnology, Ministry of Education, School of Biotechnology, Jiangnan University, Wuxi 214000, China

**Keywords:** ω-transaminases, bulky chiral amines, molecular modification, protein engineering

## Abstract

ω-Transaminases are biocatalysts capable of asymmetrically synthesizing high-value chiral amines through the reductive amination of carbonyl compounds, and they are ubiquitously distributed across diverse microorganisms. Despite their broad natural occurrence, the industrial utility of naturally occurring ω-transaminases remains constrained by their limited catalytic efficiency toward sterically bulky substrates. Over recent decades, the use of structure-guided molecular modifications, leveraging three-dimensional structures, catalytic mechanisms, and machine learning-driven predictions, has emerged as a transformative strategy to address this limitation. Notably, these advancements have unlocked unprecedented progress in the asymmetric synthesis of bulky chiral amines, which is exemplified by the industrial-scale production of sitagliptin using engineered ω-transaminases. This review systematically explores the structural and mechanistic foundations of ω-transaminase engineering. We first delineate the substrate binding regions of these enzymes, focusing on their defining features such as substrate tunnels and dual pockets. These structural elements serve as critical targets for rational design to enhance substrate promiscuity. Next, we dissect the catalytic and substrate recognition mechanisms of (*S*)- and (*R*)-ω-transaminases. Drawing on these insights, we consolidate recent advances in engineering ω-transaminases to highlight their performance in synthesizing bulky chiral amines and aim to guide future research and the industrial implementation of tailored ω-transaminases.

## 1. Introduction

In recent years, nearly 50% of the top 200 best-selling small-molecule drugs in the world contain one or more chiral amine components in their structure [1]. Synthetic methods of accessing this important type of chiral block have attracted much attention from scholars worldwide, and asymmetric reductive amination is regarded as an important synthetic approach [2,3,4]. Compared with chemical asymmetric synthesis, the asymmetric reductive amination catalyzed by ω-transaminases has the advantages of mild reaction conditions, high enantioselectivity and environmental friendliness. The theoretical yield and atomic utilization rate can both reach 100%, which is consistent with the development direction of green chemistry. Therefore, ω-transaminase-catalyzed asymmetric reductive amination has been identified as an efficient green method for preparing chiral amines [5,6]. For example, compared with the chemical synthetic route for sitagliptin, using engineered ω-transaminase from *Arthrobacter* sp. to catalyze the key chiral intermediate increased the total yield and productivity by 13% and 53% and decreased the total waste emissions by 19%. This achievement won the U.S. Presidential Green Chemistry Challenge Award in 2010 [7,8].

**Figure 1:**
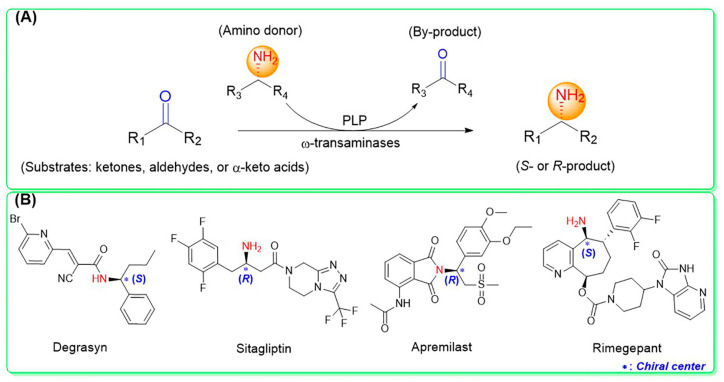
Asymmetric reductive amination catalyzed by ω-transaminases (**A**) and chiral pharmaceuticals bearing bulky substituents (**B**).

ω-Transaminases can catalyze the asymmetric reductive amination of carbonyl compounds to produce chiral amines. Distinct from other transaminases, ω-transaminases exhibit broader substrate versatility, acting not only on α-amino acids or α-keto acids but also efficiently converting ketones and aldehydes (Figure 1A). This unique capability significantly enhances their potential for industrial applications in chiral amine synthesis [9]. Investigations into the substrate specificity of ω-transaminases from microbial sources, including *Aspergillus fumigatus*, *Vibrio fluvialis*, and *Chromobacterium violaceum*, indicate that naturally occurring ω-transaminases exhibit substrate preference toward methyl ketones and relevant amines [10,11,12]. However, a critical limitation arises in pharmaceutical contexts: many bioactive chiral amines, such as the antiviral drug oseltamivir and the antidiabetic drug sitagliptin (Figure 1B), contain two sterically demanding substituents. Naturally occurring ω-transaminases struggle to accommodate such bulky substrates due to structural constraints in their substrate binding regions, severely hindering industrial utilization. Structural analyses highlight that the substrate binding region of ω-transaminases comprises a substrate tunnel and dual pockets. Spatial restrictions, particularly within the smaller pocket, which accommodates only compact substituents, render wild-type enzymes catalytically inactive or ineffective toward substrates with dual bulky groups. Overcoming this limitation by engineering ω-transaminases to expand their substrate scope remains a pivotal challenge for enabling their practical use in synthesizing pharmaceutically relevant chiral amines.

While previous reviews have addressed molecular modifications of ω-transaminases concerning substrate specificity, stereoselectivity, and stability [13], recent advancements demand renewed focus. The field has witnessed accelerated progress due to the discovery of novel ω-transaminases and breakthroughs in protein engineering technologies. In particular, structure-guided molecular modifications, leveraging three-dimensional structures, catalytic mechanisms, and machine learning-driven predictions, have emerged as pivotal strategies, especially for enabling the asymmetric synthesis of sterically demanding chiral amines. A systematic synthesis of these developments is critical to advancing the industrial application of ω-transaminases.

This review systematically explores the structural and mechanistic foundations of ω-transaminase engineering. We first delineate the substrate-binding regions of these enzymes, focusing on their defining features such as substrate tunnels and dual pockets. These structural elements serve as critical targets for rational design to enhance substrate promiscuity. Next, we dissect the catalytic and substrate recognition mechanisms of (*S*)- and (*R*)-ω-transaminases. Drawing on these insights, we consolidate recent advances in engineering ω-transaminases to highlight their performance in synthesizing bulky chiral amines. By bridging structural insights with functional outcomes, this work aims to guide future research and the industrial implementation of tailored ω-transaminases.

## 2. Structural Characteristics, Catalytic Mechanism, and Substrate Recognition Mechanism

### 2.1. Structural Classification and Substrate Binding Features

**Figure 2:**
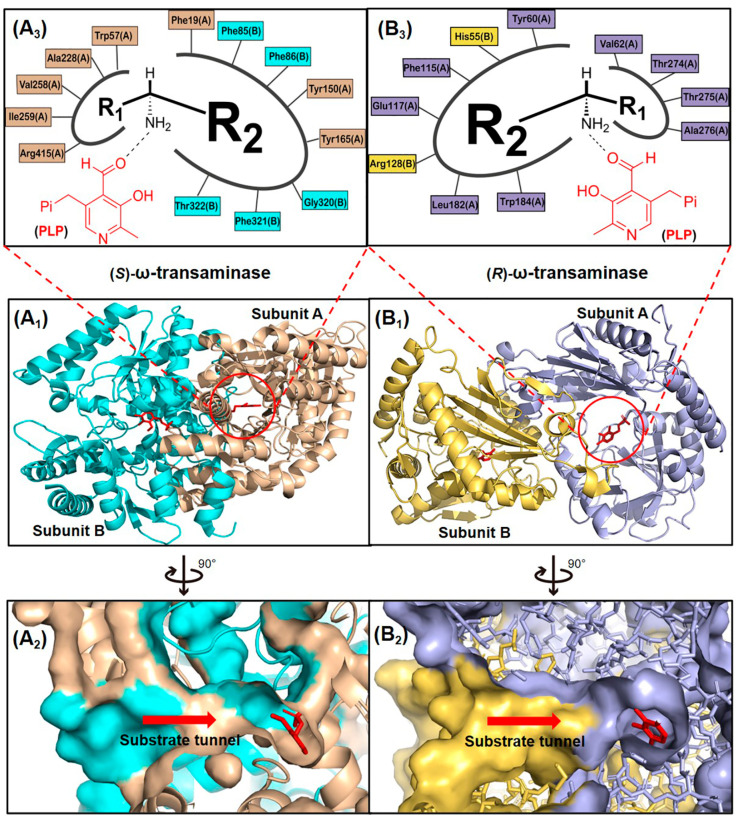
Structures of (*S*)- and (*R*)-ω-transaminases. (**A_1_**) Three-dimensional structure of (*S*)-ω-transaminase *Vf*TA with A subunit, B subunit and coenzyme PLP marked by yellow brown, cyan and red. (**B_1_**) Three-dimensional structure of (*R*)-ω-transaminase *At*TA with A subunit, B subunit and coenzyme PLP marked by blue gray, yellow and red. (**A_2_**,**B_2_**) Cross-section of substrate tunnels of *Vf*TA and *At*TA. (**A_3_**,**B_3_**) Schematic diagram of the substrate binding pockets of *Vf*TA and *At*TA, R1 and R2 representing the substituents of the chiral amine located in the small pocket and the large pocket, respectively.

ω-Transaminases represent a ubiquitous class of pyridoxal-5′-phosphate (PLP)-dependent transferases found across diverse microbial species. These enzymes are classified into two functional subgroups, (*S*)-ω-transaminases and (*R*)-ω-transaminases, based on their enantioselectivity profiles. Within the PLP-dependent enzyme superfamily, (*S*)- and (*R*)-ω-transaminases exhibit distinct structural architectures, belonging to Fold Types I and IV, respectively. Representative examples include the (*S*)-ω-transaminase from *Vibrio fluvialis* JS17 (*Vf*TA; PDB ID: 4E3Q) and the (*R*)-ω-transaminase from *Aspergillus terreus* (*At*TA; PDB ID: 4CE5), which exemplify the evolutionary divergence in fold organization and catalytic mechanisms between these subgroups [14,15]. These enzymes exhibit divergent polypeptide lengths and tertiary structural organizations, leading to both conserved catalytic motifs and pronounced architectural differences. ω-Transaminases typically function as homodimers with their active sites positioned at the subunit interface (Figure 2(A_1_,B_1_)). Substrates access the dual-pocket active site through a substrate tunnel (Figure 2(A_2_,B_2_)), where the binding cavity is partitioned into a large pocket (accommodating bulky/charged groups like aromatics or carboxylates) and a small pocket (restricted to small substituents, e.g., methyl groups, due to steric, electrostatic, and hydrophobic constraints).

In *Vf*TA, the large pocket comprises eight residues spanning both subunits: Phe19(A), Tyr150(A), Tyr165(A), Phe85(B), Phe86(B), Gly320(B), Phe321(B), and Thr322(B). The small pocket is formed by five residues from the A subunit: Trp57(A), Ala228(A), Val258(A), Ile259(A), and Arg415(A). In contrast, *At*TA features a large pocket with seven interfacial residues: Tyr60(A), Phe115(A), Glu117(A), Leu182(A), Trp184(A), His55(B), and Arg128(B), while its small pocket involves four A-subunit residues: Val62(A), Thr274(A), Thr275(A), and Ala276(A) (Figure 2(A_3_,B_3_)). The substrate access tunnels in naturally occurring ω-transaminases impose steric restrictions on bulky substrates, while the dual-pocket architecture further constrains binding efficiency. These structural features, particularly the asymmetric residue composition of the pockets, reflect evolutionary adaptations to distinct substrate profiles with fold type-dependent variations in β-sheet topology and domain packing dictating stereochemical preferences.

### 2.2. Catalytic Mechanisms

**Figure 3:**
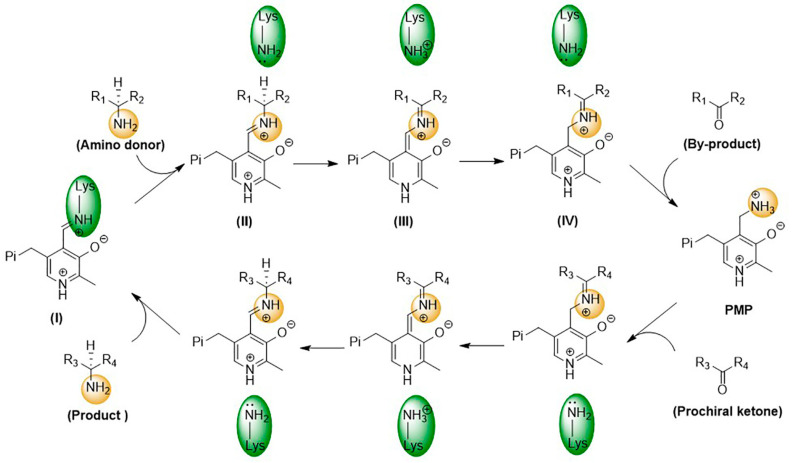
Catalytic mechanism of ω-transaminases.

As PLP-dependent transferases, ω-transaminases catalyze transamination through a two-step reversible reaction, following the ping-pong bi-bi reaction mechanism [16,17]. As shown in Figure 3, in the first step, a condensation reaction between the amino group of the catalytic residue Lys and the aldehyde group of PLP generates an internal aldehyde imide (I); then, a novel aldehyde imide (II) is formed by replacing the amino group of Lys with an external amino donor. On this basis, Lys-catalyzed proton transfer is observed to generate the quinone-type intermediate (III) and the ketoimine intermediate (IV) successively. Finally, a ketone and 5′-phosphate pyridoxamine (PMP) are generated through the hydrolysis of the ketoimine intermediate. In the second step, the amino group of PMP is transferred to the prochiral ketone to generate a chiral amine and PLP.

**Figure 4:**
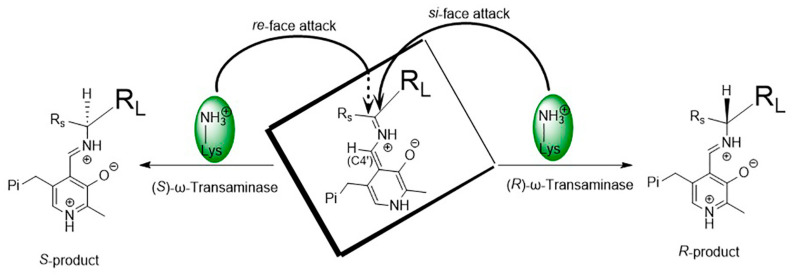
Asymmetric catalytic mechanism of ω-transaminases.

As shown in Figure 4, the spatial positions of the key catalytic residue Lys relative to PLP or the bound substrate in (*S*)-ω-transaminases and (*R*)-ω-transaminases are mirror images of each other. The stereo configuration of chiral amines, produced by the asymmetric catalysis of ω-transaminases, depends on the binding orientation of the substrate in the active center of the enzyme. In (*S*)-ω-transaminase *Vf*TA, Lys285 is located on the *si*-face of PLP (relative to C_4′_) and mainly attacks from the *re*-face of the substrate (relative to the prochiral carbon) to produce the (*S*)-product. In contrast, in (*R*)-ω-transaminase *At*TA, Lys180 in (*R*)-ω-transaminase *At*TA is located on the re-face of PLP (relative to C_4′_) and attacks mainly from the *si*-face of the substrate (relative to the prochiral carbon) to produce the (*R*)-product. In most cases, ω-transaminases show excellent stereoselectivity. Nonetheless, when substrates with special structures exhibit different binding orientations in the substrate binding pocket, chiral products with different stereo configurations are generated, thus reducing the chiral purity of the products.

### 2.3. Substrate Recognition Mechanisms

**Figure 5:**
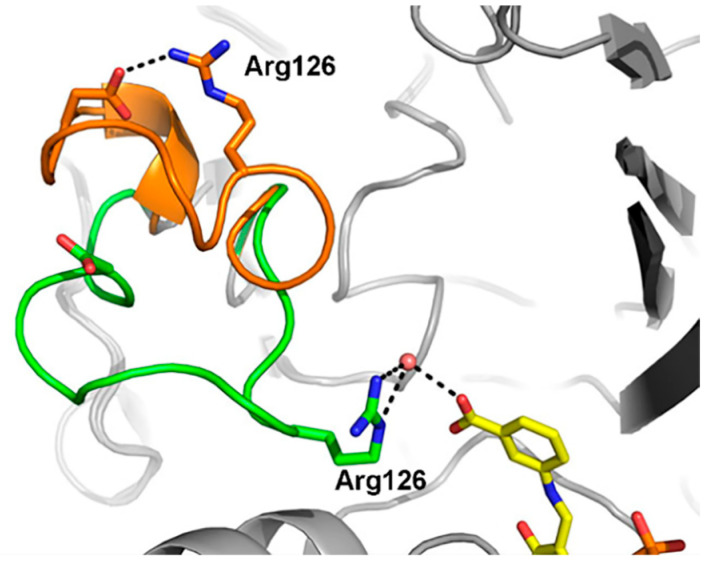
Different conformations of the loop in the substrate tunnel during the substrate recognition process of (*R*)-ω-transaminase from *Aspergillus fumigatus*. When the loop moved inward or outward, it was highlighted in green or orange accordingly [19].

In the process of amino transfer catalyzed by ω-transaminases, substrates (amino donors and ketones) with significant differences need to be bound to the enzyme successively [18]. Taking D-alanine and acetophenone as examples, the former is hydrophilic, and the latter is hydrophobic. How do ω-transaminases achieve an efficient recognition and binding of these two different substrates? Skalden and coworkers proposed the dual substrate recognition of (*R*)-ω-transaminase from *Aspergillus fumigatus*, and the substrate tunnel played a gating role in the recognition and binding of hydrophilic and hydrophobic substrates. As shown in Figure 5, the loop (green) in the substrate tunnel could be flipped, resulting in the movement of the guanidine of Arg126 into the active site to facilitate coordination to the carboxylated substrate (D-alanine). In another case, the guanidine of Arg126 could move out of the active site by flipping the loop (orange) to provide a hydrophobic environment and facilitate the binding of hydrophobic substrates (acetophenone) [19]. Dual substrate recognition has also been reported for the (*R*)-ω-transaminase from *Arthrobacter* sp., in which Arg138 plays the same important role in the substrate recognition process [20].

## 3. Molecular Modification of ω-Transaminases to Expand the Substrate Scope

### 3.1. Substrate Specificity of ω-Transaminases

Early efforts in ω-transaminase discovery primarily relied on traditional microbial culture screening methodologies. Representative examples include the isolation of *Klebsiella pneumoniae* JS2F, *Bacillus thuringiensis* JS64, and *Vibrio fluvialis* JS17 through culture screening using (*S*)-α-methylbenzylamine as the sole nitrogen source [21]. These strains demonstrated remarkable (*S*)-enantioselectivity and broad amino donor specificity toward aromatic and aliphatic chiral amines containing a methyl group adjacent to the chiral center. With the rapid development of genetic engineering, the gene sequences of multiple (S)-ω-transaminases have been reported. The (*S*)-ω-transaminase from *Vibrio fluvialis* JS17 (*Vf*TA) has been intensively studied [22]. On this basis, homologous sequence searching technology was applied to mine (*S*)-ω-transaminases of different origins, including another well-studied (*S*)-ω-transaminase from *Chromobacterium violaceum* (*Cv*TA), which showed 38% identity to *Vf*TA and also exhibited a substrate preference for methyl ketones [23]. The (*S*)-ω-transaminases mentioned above can generally catalyze the asymmetric reduction of methyl ketones to generate (*S*)-amines using L-alanine as the amino donor.

Research on (*R*)-ω-transaminases has progressed through distinct phases. (1) Pioneering discovery: In 2001, Yamada et al. reported an (*R*)-ω-transaminase from *Arthrobacter* sp. (*Ar*TA) capable of synthesizing D-alanine from pyruvate using (*R*)-α-methylbenzylamine as the amino donor. This 993 bp gene-encoded enzyme represented the first fully sequenced (R)-ω-transaminase [24]. (2) Database mining: In 2001, Höhne et al. systematically identified 17 phylogenetically diverse (*R*)-ω-transaminases through catalytic motif analysis, revealing their preferential activity toward chiral methyl-group-containing substrates like (*R*)-α-methylbenzylamine in pyruvate-driven asymmetric amination [25]. (3) Computational exploration: In 2015, Jiang et al. employed in silico motif-BLAST strategies to discover five novel (*R*)-ω-transaminases with pronounced catalytic bias for (*R*)-α-methylbenzylamine/pyruvate systems [26]. (4) Substrate scope expansion: In 2019, Telzerow et al. characterized a (*R*)-ω-transaminase from *Exophiala xenobiotica* (*Ex*TA) exhibiting native competence in converting sterically demanding substrates, including biaryl ketones, which was a notable advancement in substrate diversity [27]. To date, over thirty (*R*)-ω-transaminases have been cataloged [28,29].

Comparative analyses reveal conserved substrate preferences across both (*S*)- and (*R*)-ω-transaminases: (1) high catalytic efficiency toward methyl ketones and (2) markedly reduced or undetectable activity against substrates bearing dual bulky substituents. The restricted substrate specificity of ω-transaminases underscores the need for molecular modification to broaden substrate tolerance while preserving desired enantioselectivity.

### 3.2. Methyl Ketones and Relevant Amines Bearing One Bulky Substituent

As collated in Table 1, the molecular modification targeting methyl ketones and relevant amines bearing one bulky substituent was summarized comprehensively in this section. The catalytic activity of *Vf*TA toward aliphatic amines is usually lower than that toward aromatic amines. A mutant library of *Vf*TA was constructed using error-prone PCR by Yun and coworkers, and then the enrichment culture was carried out with 2-amino-heptane as the sole nitrogen source and 2-butanone as the inhibitor against *Vf*TA. The resulting mutant ω-Tamla P233L/V297A exhibited a 1.7- to 2-fold improvement in catalytic activity toward different aliphatic amines and a 4.5- to 6-fold improvement in the product inhibition constant toward 2-butanone and 2-heptanone [30]. On the basis of homology modeling and structure–activity relationship analysis, Cho and coworkers determined that Trp57 and Trp147 in the large binding pocket of *Vf*TA affect the binding between *Vf*TA and aliphatic amines. When Gly was introduced at these two sites, the two mutants both presented better catalytic activity toward different aliphatic amines and aromatic amines than wild-type *Vf*TA with 40.6- and 14.3-fold greater activity toward 4-phenylbutylamine and 14.6- and 1.4-fold greater activity toward 6-methylheptan-2-amine [31].

The steric hindrance effect is a key factor affecting the catalytic activity of ω-transaminases toward aromatic methyl ketones with high steric hindrance. Mutating the amino acid residues in the substrate binding region of ω-transaminases into residues with smaller side chains can effectively expand the space of the substrate binding region, thus increasing its catalytic efficiency. On the basis of in silico and in vitro studies, Dourado and coworkers designed an iterative rational strategy for reshaping the substrate binding region of *Vf*TA with a minimal number of mutations. The substrate binding region was slightly expanded by W57G/R415A mutations, which resulted in detectable activity toward 2-acetylbiphenyl, and the best mutant bearing 7 mutations presented a 1716-fold improvement in the reaction rate of the asymmetric synthesis of (1S)-1-(1,1′-biphenyl-2-yl)ethanamine [32]. The wild-type (S)-ω-transaminase from *Halomonas elongata* (*He*TA) showed detectable catalytic activity toward different aromatic methyl ketones, and the stereoelectronic effect was identified by Contente and coworkers as a crucial factor in the reaction. When the W56G mutation was introduced in the substrate binding region, a significant reduction in the stereoelectronic and steric hindrance effects was observed, resulting in a greater than 2-fold improvement in the activity toward ortho-nitroacetophenone [33]. Progress has also been made in the protein engineering of (*R*)-ω-transaminases of different origins for the catalysis of bulky biaryl ketones and naphthyl ketones. Aline and coworkers reported that naturally occurring *Ex*TA was able to convert biaryl ketones to the corresponding chiral amines and that the mutation T273S in the small binding pocket could increase the catalytic activity and broaden the substrate scope, enabling the conversion of various biaryl ketones with up to >99% conversion and excellent enantioselectivity (>99% ee) [27]. Cheng and coworkers reshaped the substrate binding pocket of (*R*)-ω-transaminase from *Mycobacterium vanbaalenii* (*Mv*TA) through a fluorescence-based high-throughput screening method to increase its catalytic activity toward bulky naphthyl ketones. Two beneficial substitutions (G68Y and F129A) and three improved mutants were identified from approximately 8000 clones, among which the best mutant, *Mv*TA-M5, exhibited more than a 100-fold improvement in activity toward acetonaphthone with ee > 99% (*R*) [34].

Although ω-transaminases exhibit a relatively broad substrate spectrum, they generally have much lower catalytic activity toward ketones and aldehydes than toward their natural substrate pyruvate, which limits their application in the asymmetric synthesis of chiral amines. To improve the substrate preference of (*S*)-ω-transaminase from *Ochrobactrum anthropic* (*Oa*TA), eight amino acid residues in the active center (<3 Å) were selected for alanine scanning, resulting in a positive mutation, W58A, that improved the catalytic activity. The final variant W58L was further identified via saturation mutagenesis and exhibited a 41- to 760-fold improvement in activity toward different methyl ketones and a 59% decrease in catalytic activity toward pyruvate [35]. On the basis of homologous sequence alignment and protein structure analysis, four amino acid residues (Leu56, Trp57, Arg415, and Leu417) in the large binding pocket of *Vf*TA were shown to play important roles in the binding of *Vf*TA to α-keto acids or aldehydes. The substrate preference of *Vf*TA was converted from pyruvate to pentanal when certain mutations were introduced at these four sites. When (*S*)-1-phenylethylamine, (*S*)-1-phenylpropylamine, or (*S*)-1-phenylbutylamine was used as the amino donor, the best mutant, M3 (W57F/R415L/L417V), exhibited 2.8-, 3.0-, and 5.1-fold increases in activity toward pentanal, respectively [36]. In our previous research, (*R*)-ω-transaminase *At*TA was found to exhibit much lower catalytic activity toward 4-hydroxy-2-butanone than toward pyruvate. The substrate tunnel and binding pocket were reshaped by a combinatorial active-site saturation test, resulting in the best mutant *At*TA-M5 (H55A/G126F/S215P) with significantly improved catalytic efficiency and thermal stability for the asymmetric synthesis of (*R*)-3-amino-1-butanol. Moreover, the ability of the key mutation S215P to improve the catalytic efficiency of seven other homologous (*R*)-ω-transaminases was validated [37]. Ketones and aldehydes have always been important raw materials for the asymmetric synthesis of chiral amines, and improving the catalytic ability of ω-transaminases toward these compounds could effectively promote their industrial application.

**Table 1:** Molecular modification targeting methyl ketones and relevant amines bearing one bulky substituent.

Enzymes	Substrates or Products	Key Mutations	References
*Vf*TA	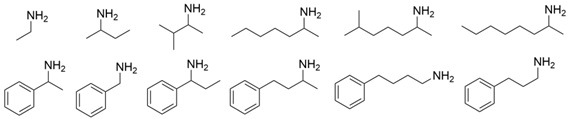	P233L/V297A	[30]
	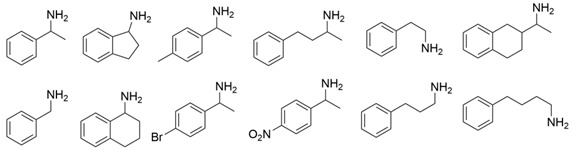	W57G and W147G	[31]
	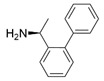	W57G/R415A	[32]
	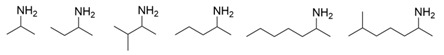	W57F/R415L/L417V	[36]
*He*TA	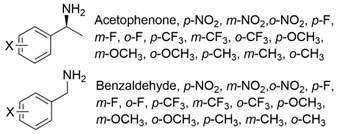	W56G, Y149F, F84A, and I258A	[33]
*Ex*TA	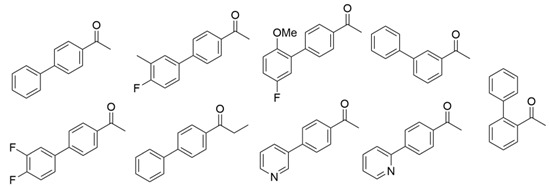	T273S and K110R/L191F/N249F/E300K/K317E	[27]
*Mv*TA	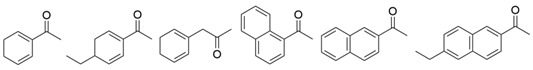	G68Y/F129A	[34]
*Oa*TA	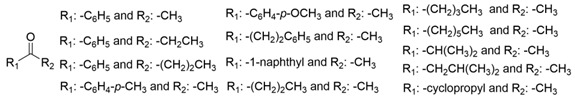	W58A and W58L	[35]
*At*TA	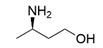	H55A/G126F/S215P	[37]

### 3.3. Ketones and Relevant Amines Bearing Two Bulky Substituents

In most pharmaceutical chiral amines, there are two bulky substituents on both sides of the chiral carbon atom. Naturally occurring ω-transaminases show negligible or undetectable ability to synthesize these compounds, which has become the main bottleneck restricting the application of ω-transaminases in the asymmetric synthesis of chiral amines. As collated in Table 2, the molecular modification of ω-transaminases targeting the substrate tunnel and binding pocket was able to address this challenge effectively, thus expanded the application scope of ω-transaminases in chiral amine synthesis.

Among ω-transaminases of different origins, the molecular modification of *Vf*TA to facilitate the asymmetric catalysis of bulky substrates has been reported extensively. Owing to the initial lack of clear information about the three-dimensional structure and catalytic mechanism of *Vf*TA, the directed evolution strategy was employed. Error-prone PCR was used by Hwang and coworkers for random mutation of the target gene, and high-throughput screening was subsequently carried out by using 3-amino-3-phenylpropionic acid as the sole nitrogen source for primary screening culture and observing the appearance of color generated by the reaction of α-amino acids and copper ions. The resulting mutants exhibited up to a 3.1-fold improvement in the conversion of 3-amino-3-phenylpropionic acid [38]. With extensive studies on the structure and mechanism, structure-dependent rational design and mechanism-guided computational design methods have been successfully employed to engineer *Vf*TA to improve its catalytic ability toward bulky substrates. In the protein engineering of *Vf*TA for the synthesis of (3*S*,5*R*)-3-amino-5-methyl-octanoate ethyl ester from (*R*)-5-methyl-3-oxo-octanoate ethyl ester, homologous sequence alignment and ProteinGPS^TM^ statistical analysis were combined to construct approximately 450 mutants, and the best mutant, r414, bearing eight mutations, showed more than 60-fold improved catalytic activity. To further improve protein engineering efficiency, a computational protocol for predicting the catalytic activity of *Vf*TA mutants was developed by Sirin and coworkers via the combination of molecular dynamics, docking, and MM-GBSA scoring. Eighty-nine mutants were constructed by using this protocol, including the best mutant, r414, which was mentioned above. Two optimal mutants were then identified with 20- and 60-fold improvements in catalytic activity [39]. Naturally occurring *Vf*TA showed negligible catalytic activity toward α-hydroxy ketones and aryl-alkyl ketones bearing an alkyl substituent larger than a methyl group. Systematic mutagenesis of the active site residues was carried out by Nobili and coworkers to expand the substrate scope. Two mutants, F85L/V153A and Y150M/V153A, were subsequently identified, with a 26-fold increase in activity toward (*S*)-phenylbutylamine and a 53-fold increase in activity toward (*R*)-phenylglycinol, respectively [40]. In addition, *Vf*TA has been applied to synthesize other chiral amines bearing two bulky substituents. Genz and coworkers selected seven residues of *Vf*TA through bioinformatic tools to expand the substrate binding pocket, which was followed by mutant library construction and high-throughput screening. Three mutants allowing the asymmetric synthesis of tert-butylphenyl amine were identified, among which the best mutant, bearing four mutations (L56V, W57C, F85V, V153A), presented high catalytic activity and stereoselectivity in the asymmetric synthesis of (*R*)-2,2-dimethyl-1-phenylpropan-1-amine [41]. (2*R*,4*S*)-5-([1,1′-biphenyl]-4-yl)-4-amino-2-methylpentanoic acid, a chiral precursor of the heart failure drug sacubitril, is very difficult to synthesize asymmetrically by using naturally occurring ω-transaminases. Using the Codex Amine Transaminase Screening Kit, Scott and coworkers identified a variant of *Vf*TA ATA-217 bearing seventeen mutations, which exhibited trace activity and preferences for the undesired diastereoisomer. Through eleven rounds of enzyme evolution, the new variant CDX-043 was obtained. CDX-043 showed high productivity, excellent stereoselectivity, and high process robustness and allowed the efficient and cost-effective production of sacubitril at a large scale [42].

Another well-studied (*S*)-ω-transaminase, *Cv*TA, was characterized by Kaulmann and coworkers in 2007. *Cv*TA has high potential as a useful biocatalyst because of its broad amino acceptor range but lacks the ability to accommodate substrates with bulky groups on both sides of the chiral or prochiral carbon. The residues in the small binding pocket and substrate tunnel have been successively targeted to expand the substrate scope of *Cv*TA toward sterically demanding substrates. Two double mutants (F88L/C418(G/L)) developed by Moritz Voss and coworkers showed more than 200-fold improved activity in the conversion of (*S*)-1-phenylbutylamine [43]. Another double mutant (L59A/F88A) was successfully applied in the kinetic resolution of 1,2-diphenylethylamine, generating enantiomerically pure (*R*)-1,2-diphenylethylamine with ee > 99% [44]. Owing to the enlarged small binding pocket and good tolerance to high concentrations of isopropylamine, the single mutant (L59A) of *Cv*TA was also employed for the asymmetric synthesis of (*S*)-1-phenylbutylamine, producing bulky amines in enantiomerically pure form (>99% ee) [45]. For the key chiral intermediate of rimegepant with two rigid and sterically hindered substituents, naturally occurring ω-transaminases with detectable activity toward the desired ketone have not yet been identified. Ma and coworkers proposed a two-pronged strategy to access this asymmetric synthesis. First, a variant derived from *Cv*TA was identified by screening the transaminase library using a truncated substrate analog and selected as the starting scaffold. A combination of a rational approach and several evolution strategies was subsequently carried out to enlarge the substrate binding pocket. Through ten rounds of enzyme evolution, the resultant variant showed more than 50,000-fold improved activity and high stereoselectivity (>99.5% de), which indicated promise for industrial application [46]. For substrates with greater steric hindrance and complex structures, screening of the starting scaffold and the further rational design of naturally occurring enzymes via a truncated substrate analog constitute a recommended strategy, which was reported earlier in the biocatalytic asymmetric synthesis of the sitagliptin intermediate by Codexis and Merck. Owing to the greater steric hindrance of the prositagliptin ketone, none of the naturally occurring ω-transaminases with detectable activity were screened. A substrate walking approach was first applied to create a single mutant S223P of (*R*)-ω-transaminase *Ar*TA, which exhibited catalytic activity toward the truncated methyl ketone analog. Another mutant, S223P/Y26H/V65A/V69G/F122I/A284G, was subsequently developed via directed evolution and rational design; this mutant exhibited catalytic activity toward bulky prositagliptin ketones for the first time. Through eleven rounds of enzyme evolution, the final mutant Rd11TA, featuring twenty-seven mutations, exhibited more than 28000-fold improved catalytic activity and was successfully applied in the manufacture of sitagliptin [7,47].

In addition to *Vf*TA and *Cv*TA, studies on the molecular modification of ω-transaminases of other origins have also been extensively reported. (*S*)-ω-transaminases from *Caulobacter crescentus* (*Cc*TA) and *Burkholderia vietnamiensis* (*Bv*TA) were engineered to accept bulky β-amino acids and esters, respectively. When Val227 and Asn285 in the substrate binding pocket of *Cc*TA were changed to Gly and Ala, which have smaller side chains, the catalytic activity toward 3-amino-3-phenylpropionic acid increased 1.4-fold and 1.9-fold, respectively, whereas the catalytic activity toward 3-aminobutyric acid decreased to 56% and 28%, respectively, of that of wild-type *Cc*TA [48]. *Bv*TA showed poor catalytic activity toward β-keto esters with little steric hindrance and was further engineered to accept bulky β-keto esters by Wang and coworkers. The best mutant, HBV_MIII, was applied in the asymmetric synthesis of aliphatic β-amino acids at the semipreparative scale with high yield and enantioselectivity [49].

α-Keto acids are favorable substrates for ω-transaminases in the production of enantiopure amino acids, but the naturally occurring enzymes show little or no reactivity toward α-keto acids bearing larger substituents. Park and coworkers reported the first example in which a naturally occurring (*S*)-ω-transaminase from *Paracoccus denitrificans* (*Pd*TA) was able to accommodate the bulky n-butyl substituent in the small binding pocket. Alanine scanning mutagenesis was then applied to six different residues of the small binding pocket, which revealed that the V153A mutant exhibited significant catalytic activity toward α-keto acids bearing bulky n-hexyl substituents [50]. (*R*)-ω-transaminase from *Gibberella zeae* (*Gz*TA) was also selected as a starting scaffold because of its high activity and stereoselectivity. By reshaping the substrate tunnel and binding pocket, the conversion activity of the triple mutant F113L/V60A/S214A toward 2-oxo-2-phenylacetic acid increased by 3.8-fold relative to that of the wild-type enzyme [51].

Owing to the special structure of the substrate tunnel and binding pocket, chiral aryl alkyl amines with a small substituent larger than the methyl group have always been difficult to synthesize using naturally occurring ω-transaminases. Han and coworkers reported that the orientation of the bound prochiral ketone in the (*S*)-ω-transaminase from *Ochrobactrum anthropic* (*Oa*TA) was not conducive to nucleophilic attack during the reaction. An efficient computational strategy was then developed to create high-turnover mutants tailored for a target ketone, resulting in a double mutant, L57A/W58A, with 105-fold activity improvement toward butyrophenone and no loss of stereoselectivity [52]. Konia and coworkers engineered (*R*)-ω-transaminase from *Luminiphilus syltensis* (*Ls*TA) to expand the small binding pocket, and the V37A mutant exhibited increased activity toward 1-phenylpropylamine and 1-butylamine [53]. Instead of modifying the small binding pocket, Xie and coworkers rationally engineered the large binding pocket of an (*S*)-ω-transaminase from *Paraburkholderia phymatum* (*Pp*TA) to relieve the inherent restriction caused by the small binding pocket. The resulting quadruple mutant M78F/W82A/I284F/T440Q exhibited 104- to 230-fold improved activity toward various bulky aryl alkyl ketones with a small substituent bulkier than the methyl group [54].

Chiral cyclic and bicyclic amines have also been asymmetrically synthesized using engineered ω-transaminases, whereas naturally occurring enzymes result in poor productivity for these targets. Martin and coworkers reported the directed evolution of (*S*)-ω-transaminase from *Athrobacter citreus* (*Ac*TA) using error-prone PCR. When substituted (*S*)-aminotetralin and pyruvate were selected as the substrates, the resulting substituted tetralone was colored upon exposure to air. The selection of the mutants with improved properties was based on the appearance of color. Through five rounds of enzyme evolution, a final mutant, bearing seventeen mutations, was obtained and exhibited 268-fold improved activity with reduced biocatalyst loading and an overall improvement in product yield [55]. (*S*)-ω-transaminase from *Pseudomonas jessenii* (*Pj*TA) showed no detectable activity for the synthesis of six bicyclic or bulky amines, and a protein engineering strategy using computational design was explored by Meng and coworkers to expand the substrate scope. The single mutant W58G and triple mutant W58M/F86L/R417L demonstrated superior catalytic performance in the asymmetric synthesis of chiral bicyclic or bulky amines, with the former showing optimal efficiency for indane- and tetralin-containing substrates, while the latter exhibited enhanced activity toward 1-phenylbutylamine [56]. (*R*)-ω-transaminase from *Rhodobacter* sp. (*Rh*TA) was redesigned by Li and coworkers for the asymmetric synthesis of chiral N-heterocyclic amines. Through combined mutation, the quadruple mutant RbTAM2 was developed and exhibited significantly improved activity (4- to 1064-fold) toward a series of cyclic and bulky heterocyclic ketones [57].

Owing to the structural differences among ω-transaminases of different origins, the protein engineering of individual enzymes has always been difficult. In view of this, a systematic study should be carried out on the structure–activity relationship of ω-transaminases for the catalysis of bulky substrates, which will help to promote the molecular modification of ω-transaminases for the asymmetric synthesis of bulky amines. An intensive study of (*S*)-ω-transaminase from *Ruegeria* sp. TM1040 (3FCR) in the asymmetric catalysis of bulky substrates was reported by Bornscheuer and coworkers. The 3FCR mutants were developed by extensive protein engineering followed by optimization of the identified motif, producing mutants with 8900-fold improvements in activity and high stereoselectivity in the asymmetric synthesis of several chiral amines bearing two bulky substituents. The identified motif (Y59W/Y87F/Y152F/T231A) has been demonstrated to be generally applicable in six other new (*S*)-ω-transaminases [58]. The quadruple mutant 3FCR_QM obtained in the above research was then selected as the starting scaffold for the asymmetric synthesis of a bicyclic and bridged amine. Five positive mutants were developed through protein engineering combining rational design and directed evolution, the conversion of which was improved 2.2–4.2-fold over that of 3FCR_QM in the asymmetric synthesis of exo-3-amino-8-aza-bicyclo[3.2.1]oct-8-yl-phenyl-methanone [59]. Another mutant, 3FCR_WLAMA (Y59W, Y87L, T231A, L382M, and G429A), was developed through the same strategy and can perform the asymmetric synthesis of bulky (*R*)-2,2-dimethyl-1-phenylpropan-1-amine with high conversion [60]. Recently, machine learning has been employed to provide a promising approach for the protein engineering of 3FCR by the above research team. A number of 3FCR mutants were developed via directed evolution and rational design, and their activity was then determined to generate a high-quality dataset for building a machine learning model. This model was applied for the data-driven design of optimized 3FCR mutants with improved activity toward different bulky substrates and the prediction of catalytic activity for the mutants of another (*S*)-ω-transaminase 3HMU by retraining with a small set of additional data [61,62].

**Table 2:** Molecular modification targeting ketones and relevant amines bearing two bulky substituents.

**Enzymes**	Substrates or Products	Key Mutations	References
*Vf*TA	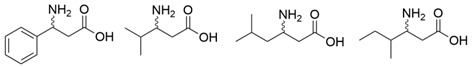	Not mentioned	[38]
	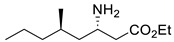	F19W/W57F/F85A/R88K/V153A/K163F/I259V/R415F	[39]
	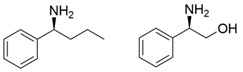	F85L/V153A Y150M/V153A	[40]
	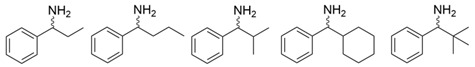	L56V/W57C/F85V/V153A	[41]
	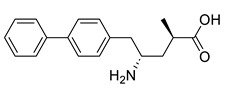	ATA217+Y17M/F19H/V31M/V42F/N53C/L57A/K66P/F85V/S86N/R146H/Y165W/R203H/C260T/S284A/S286G/F291Y/P293A/A313L/I314R/E316W/G320A/G394P/C414V/P416A/V422A/C424A	[42]
*Cv*TA	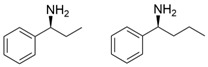	F88L/C418G F88L/C418L	[43]
	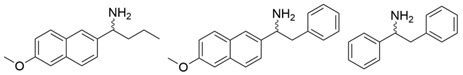	L59A/F88A	[44]
	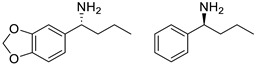	L59A	[45]
	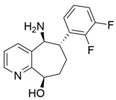	L59A/F88A/V234A/L380A/Y89D/N86H/Y85M/T91I/P83S/K90G/S417I/S424A/F301S/G164S/T452S/M180V/F449L/F320H/Y322T	[46]
*Ar*TA	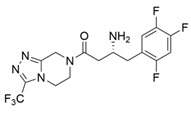	S8P/Y60F/L61Y/H62T/V65A/V69T/D81G/M94I/I96L/F122M/S124/S126T/G136F/Y150S/V152C/A169L/V199I/A209L/G215C/G217N/S223P/L269P/L273/T282S/A284G/P297S/V306I/S321P	[7]
*Cc*TA	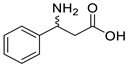	V227G and N285A	[48]
*Bv*TA	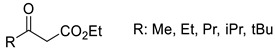	L57A/W58F/F86M/A154S/I260V	[49]
*Pd*TA	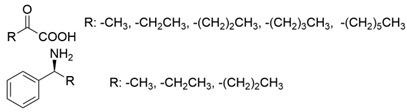	V153A	[50]
*Gz*TA	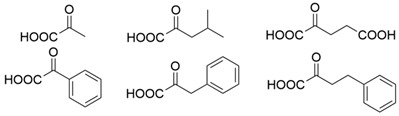	F113L/V60A/S214A	[51]
*Oa*TA	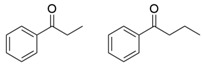	L57A/W58A	[52]
*Ls*TA	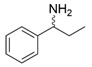	V37A	[53]
*Pp*TA	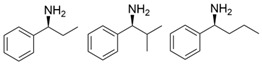	M78F/W82A/I284F/T440Q	[54]
*Ac*TA	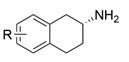	M46T/D48G/Y60C/Y164F/Y185C/N186S/P195S/M197T/C205Y/A242V/A245T/I252V/F255I/N268S/T409R/K424E/V436A	[55]
*Pj*TA	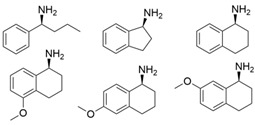	W58G and W58M/F86L/R417L	[56]
*Rh*TA	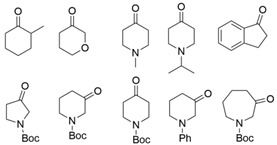	Y125A/I6A/L7A/L158V	[57]
3FCR	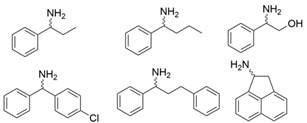	Y59W/Y87F/Y152F/T231A	[58]
	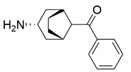	Y59L/S86A/Y87F/Y152F/T231A/I234M/L382M	[59]
	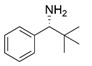	Y59W/Y87L/T231A/L382M/G429A	[60]
	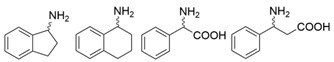	Y59W/Y87F/T231A/S19W Y59W/Y87F/T231A/Y152F F91F/T231A/Y59W F91L/S19W/T231L/Y59W	[61]
	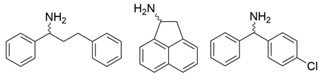	Y59W/Y87F/T231A/S155A Y59W/Y87F/T231A/F167Y Y59W/Y87F/T231A/F168W Y59W/Y87L/T231A/Y152F Y59W/Y87F/T231A/Y152F/L382M Y59W/Y87F/T231A/Y152F/F168W	[62]

## 4. Conclusions

The molecular modification of ω-transaminases has achieved remarkable advancements through precise elucidation of their three-dimensional structures and catalytic mechanisms, particularly in enabling the asymmetric reductive amination of sterically demanding substrates. This field has witnessed a paradigm shift from traditional random mutagenesis approaches to structure-guided rational engineering strategies facilitated by emerging computational biology tools and structure–function relationship analyses. Such progress has significantly enhanced the precision and efficiency of ω-transaminases optimization. Concurrently, the substrate focus has evolved from simple methyl ketones to structurally complex, high-value pharmaceutical prochiral ketones, substantially expanding their potential in sustainable biomanufacturing. However, two critical challenges persist in practical implementation: (1) the pronounced substrate specificity of ω-transaminases creates substantial limitations in processing structurally diverse compounds, and (2) the delayed exploration and limited availability of (*R*)-ω-transaminases compared to their (*S*)-counterparts have severely constrained the synthesis of chiral amines and particularly crucial (*R*)-configured pharmaceutical amines. Furthermore, current laboratory-scale achievements often fail to meet industrial requirements due to insufficient catalytic efficiency, substrate tolerance, and operational stability under process conditions.

To address these challenges, future research priorities should focus on the following: (1) developing innovative enzyme engineering strategies combining computational design and directed evolution to broaden the substrate scope, (2) establishing high-throughput screening platforms for discovering novel ω-transaminases, and (3) enhancing biocatalyst robustness through immobilization techniques and reaction engineering. These advancements will be essential to bridge the gap between academic research and industrial implementation, ultimately advancing ω-transaminases as versatile biocatalysts in green chemistry applications.

## Data Availability

No new data were created or analyzed in this study.

## Abbreviations

The following abbreviations are used in this manuscript:

PLP	pyridoxal-5′-phosphate
*Vf*TA	(*S*)-ω-transaminase from *Vibrio fluvialis* JS17
*Cv*TA	(*S*)-ω-transaminase from *Chromobacterium violaceum*
*At*TA	(*R*)-ω-transaminase from *Aspergillus terreus*
*Ex*TA	(*R*)-ω-transaminase from *Exophiala xenobiotica*
*Ar*TA	(*R*)-ω-transaminase from *Arthrobacter* sp.
*He*TA	(S)-ω-transaminase from *Halomonas elongata*
*Mv*TA	(*R*)-ω-transaminase from *Mycobacterium vanbaalenii*
*Oa*TA	(*S*)-ω-transaminase from *Ochrobactrum anthropic*
*Cc*TA	(*S*)-ω-transaminase from *Caulobacter crescentus*
*Bv*TA	(*S*)-ω-transaminase from *Burkholderia vietnamiensis*
*Pd*TA	(*S*)-ω-transaminase from *Paracoccus denitrificans*
*Gz*TA	(*R*)-ω-transaminase from *Gibberella zeae*
*Oa*TA	(*S*)-ω-transaminase from *Ochrobactrum anthropic*
*Ls*TA	(*R*)-ω-transaminase from *Luminiphilus syltensis*
*Pp*TA	(*S*)-ω-transaminase from *Paraburkholderia phymatum*
*Ac*TA	(*S*)-ω-transaminase from *Athrobacter citreus*
*Pj*TA	(*S*)-ω-transaminase from *Pseudomonas jessenii*
*Rh*TA	(*R*)-ω-transaminase from *Rhodobacter* sp.
